# Mechanistic Study on Orpiment Pigment Discoloration Induced by Reactive Oxygen Species

**DOI:** 10.3390/molecules30163318

**Published:** 2025-08-08

**Authors:** Jiaxing Sun, Zhehan Zhang, Xiaofen Chen, Qin Huang, Zhilin Bian, Wenyuan Zhang, Bomin Su, Haixia Zhang

**Affiliations:** 1 State Key Laboratory of Natural Product Chemistry, College of Chemistry and Chemical Engineering, Lanzhou University, Lanzhou 730000, China; sunjx2021@lzu.edu.cn (J.S.); zhangzhh2024@lzu.edu.cn (Z.Z.); chenxiaofen@lzu.edu.cn (X.C.); hqin2023@lzu.edu.cn (Q.H.); 320220920560@lzu.edu.cn (Z.B.); 2 Gansu Provincial Research Center for Conservation of Dunhuang Cultural Heritage, Dunhuang Academy, Dunhuang 736200, China; zhangwy@dha.ac.cn (W.Z.); subm@dha.ac.cn (B.S.)

**Keywords:** orpiment, reactive oxygen species, discoloration

## Abstract

Orpiment (As_2_S_3_), a yellow mineral pigment widely used in historical artworks, undergoes degradation that seriously threatens the integrity of Dunhuang murals. Reactive oxygen species (ROS) exist widely in air, which may be one reason for the color change of pigments. This study aims to investigate the degradation effects and mechanisms of four ROS—hydroxyl radical (·OH), singlet oxygen (^1^O_2_), peroxynitrite anion (ONOO^−^), and hydrogen peroxide (H_2_O_2_)—on orpiment. By simulating chemical reaction systems, the interaction processes between different ROS and orpiment were qualitatively and quantitatively analyzed, and the degradation capacities of each ROS on orpiment were evaluated. The experiments show that all ROS can induce orpiment degradation, among which ·OH exhibits the strongest degradation capacity due to its high oxidation potential, while ^1^O_2_, ONOO^−^, and H_2_O_2_ have relatively minor impacts on orpiment aging. It is the first time that a study has confirmed that ROS (especially ·OH) may drive orpiment degradation in environments, contributing to the increasing number of conservation strategies for artworks.

## 1. Introduction

Orpiment (As_2_S_3_) is a primary mineral-based pigment, whose usage can be traced back to around 3000 BCE in ancient Egypt and ancient China. As an important yellow pigment, orpiment has been widely applied in various art forms worldwide, including murals, oil paintings, manuscripts, etc. [1]. Over time, the orpiment layers on the surface of most artworks have undergone different degrees of degradation, showing phenomena such as fading, darkening, flaking, and pulverization. Many cultural heritage conservators have studied this aging. Traditional studies have mostly focused on how to avoid or mitigate the further degradation of orpiment, as well as analyzing its photodegradation products via spectroscopy [2,3,4,5,6,7]. However, there are still blind spots in explaining the photodegradation mechanism of orpiment. The ambiguity of this key mechanism has led existing protection strategies to remain at an empirical level. Therefore, in-depth analysis of its photodegradation mechanism is imperative to provide theoretical support for the protection of pigment layers.

Keyser et al. [8] investigated the fading phenomenon of yellow roses in oil paintings (where the main pigment is orpiment and red lead), identified the spatial distribution of orpiment and its degradation products (such as As_2_O_3_, PbHAsO_4_, Pb_5_(AsO_4_)_3_Cl, etc.), and analyzed the elemental distribution in the pigment cross-section. They found the migration and precipitation of lead arsenate (Pb_3_(AsO_4_)_2_) in pigment layers, proposing that As_2_O_3_ generated by orpiment degradation further reacted with lead ions to form precipitates, causing fading and pulverization of yellow rose. This reveals the irreversible impact of arsenic-based pigment degradation on the optical effects of the oil paintings. Keune et al. [9] analyzed multiple paintings and samples containing pigments such as orpiment, realgar, and Paris green (Cu(C_2_H_3_O_2_)_2_·3Cu(AsO_2_)_2_), discovering that As elements were partly soluble and migrated within the paintings after arsenic-containing pigment degradation, and As(V) can be detected from the substrate to the surface. Based on this, they proposed that orpiment degradation first formed As_2_O_3_, which was partly dissolved in water and further oxidized to As_2_O_5_, which reacted with lead, calcium, and other ions to generate insoluble arsenates. Broers et al. [10] proposed two degradation routes of orpiment in oil paintings: under dry conditions, orpiment powder formed only As_2_O_3_ upon illumination; when orpiment was mixed with water-containing binders (such as egg white) and illuminated, it produced As(Ⅴ) compounds, which migrated and further reacted with environmental cations to form insoluble metal arsenates. The stress generated during this process led to cracks and delamination in artworks. Vermeulen et al. [11] mixed orpiment with pigments such as basic copper acetate (Cu(OH)_2_·(CH_3_COO)_2_·5H_2_O) and red lead (Pb_3_O_4_), subjected them to light aging, and found that with time, accompanied by the release of H_2_S, Cu_2_S, and PbS, appeared in the mixed pigments. Based on this phenomenon, they proposed that during the photodegradation of orpiment, structural rearrangement occurs with the release of sulfur atoms, which are speculated to be sulfur radicals. These released sulfur atoms can react with surrounding substances in the environment, thereby generating H_2_S, which affects copper- or lead-containing pigments to form black Cu_2_S or PbS. Previous studies have predominantly focused on the degradation products of orpiment and the migration behavior of these products, while systematic investigations into the specific degradation mechanisms remain scarce.

Atmospheric photochemistry is the core mechanism for the generation of free radicals in the atmosphere, producing highly reactive atoms and transient free radicals through the interaction of sunlight with photolyzable molecules (such as ozone and nitrogen oxides). Although the concentrations of these transient free radicals in the atmosphere are extremely low (average concentration about 10^−15^–10^−12^ mol·L^−1^), their high reactivity makes them key mediators for regulating atmospheric components [12]. Among them, the hydroxyl radical (·OH), as the main oxidant, can reach a daytime concentration of approximately 10^6^ molecules/cm^3^, reacting with trace pollutants and greenhouse gases, making it crucial for pollutant removal [13]. Reactive oxygen species (ROS) such as hydroxyl radicals, hydrogen peroxide (H_2_O_2_), singlet oxygen (^1^O_2_), and peroxynitrite anions (ONOO^−^) are easily generated under ultraviolet and high-oxygen conditions, and their high oxidizing properties may accelerate the degradation of substances in contact with them.

Based on the hypothesis that the photodegradation of orpiment is essentially caused by ROS generated through atmospheric photochemistry, this study designed and validated the degradation process of orpiment in the presence of ROS, characterized the reaction products, and confirmed the degradation capability of ROS toward orpiment. The specific research contents are as follows: according to the weakly alkaline characteristics of Dunhuang mural walls, reactions of orpiment with various ROS were designed under near-neutral pH conditions. ROS species capable of reacting with orpiment—including ·OH, ^1^O_2_, ONOO^−^, and H_2_O_2_—were screened out. Control experiments were conducted to eliminate the interference of single reagents, verify the efficient degradation capacity of ROS in relation to orpiment, and compare the degradation effects of different ROS species. By characterizing the solutions and precipitates at different reaction times with various ROS, the reaction products and corresponding chemical equations were explored.

## 2. Results

### 2.1. Verification of the Reactivity of Four Kinds of Reactive Oxygen Species

**Figure 1:**
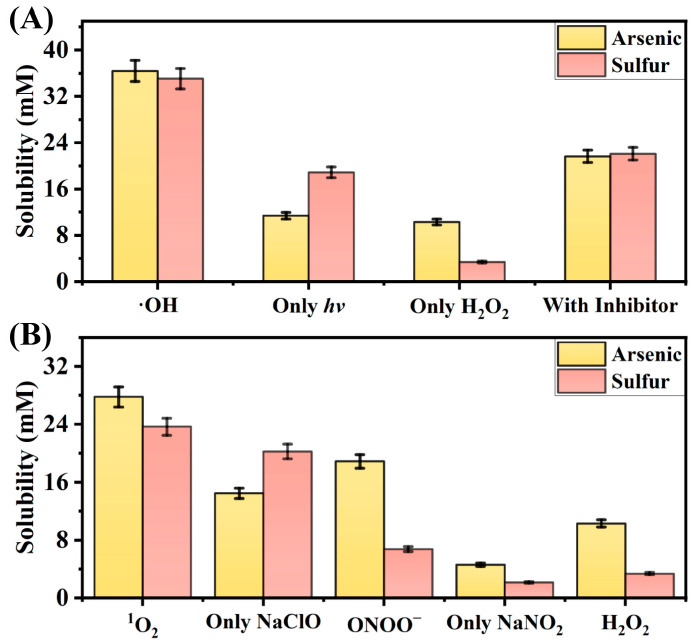
Total arsenic and sulfur contents in the solutions of the (**A**) ·OH, (**B**) ^1^O_2_, ONOO^−^, and H_2_O_2_ experimental groups and the control group after 6 h of reaction measured by ICP-OES.

First, the experiments were conducted to study the reactions between four common ROS in the atmosphere, namely, ·OH, ^1^O_2_, ONOO^−^, or H_2_O_2_, and orpiment. ICP-OES was used to analyze the supernatants collected after 6 h of reaction, determining the total arsenic and sulfur contents in each group. As shown in Figure 1, the dissolved amounts of As and S in the experimental groups (ROS groups) were significantly higher than those in the control group, confirming that ROS can degrade orpiment. After a 6 h reaction of orpiment with ·OH, approximately 65% of orpiment degrades into water-soluble ions. Notably, the As:S molar ratio in As_2_S_3_ is 2:3, whereas the ratio in the dissolved species is approximately 1:1, indicating that part of the sulfur may convert to insoluble sulfur compounds (e.g., elemental sulfur). Comparing the reaction results of four reactive oxygen species (·OH, ^1^O_2_, ONOO^−^, H_2_O_2_), the As and S dissolution ratio decreases in an order consistent with their oxidation potentials—i.e., the higher the oxidation potential, the stronger the degradation capacity of the reactive oxygen species toward orpiment.

**Figure 2:**
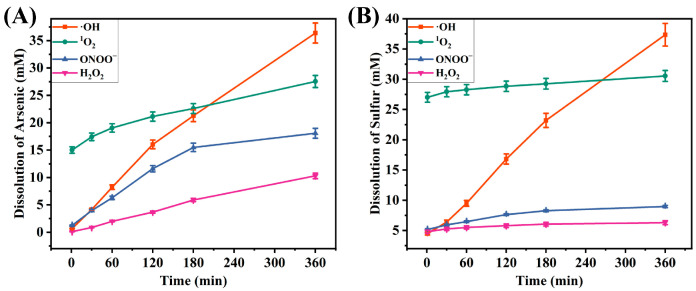
Curves of arsenic (**A**) and sulfur (**B**) concentrations in supernatants as a function of time in different ROS systems.

To systematically explore the differences in the degradation efficiencies of the four ROS toward As_2_S_3_, quantitative analysis of the reaction system was carried out using ICP-OES. By measuring the dissolution amounts of arsenic and sulfur at different time points, their dynamic dissolution trends were obtained. As depicted in Figure 2, the experimental data show that the ·OH significantly outperforms ^1^O_2_, ONOO^−^, and H_2_O_2_ in both degradation rate and total degradation amount, demonstrating excellent degradation activity. A significant linear trend was observed during the degradation of orpiment by ·OH, indicating favorable kinetic characteristics of the reaction. It is worth noting that in the ^1^O_2_ experimental group, a sharp increase in the dissolution of arsenic and sulfur elements was observed at the initial stage of the reaction (1 min). The analysis indicates that this phenomenon can be attributed to sodium hypochlorite, a strongly oxidizing reagent used in the preparation of ^1^O_2_. Its oxidizing effect accelerates the decomposition of As_2_S_3_, thus leading to the rapid dissolution of arsenic and sulfur elements at the initial stage.

### 2.2. Characterization of the Reaction Results of ·OH

**Figure 3:**
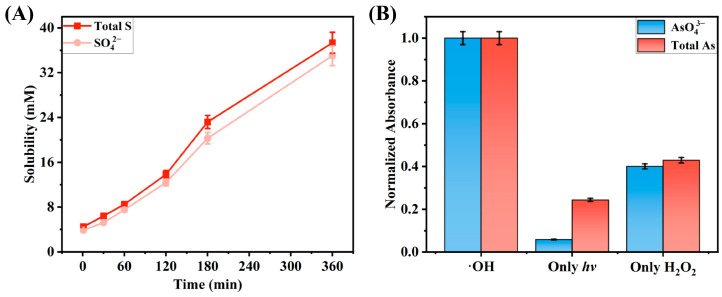
Characterization of the solution after the reaction between ·OH and As_2_S_3_: (**A**) trends of total S and SO_4_^2−^ in the solution over time; (**B**) proportion of AsO_4_^3−^ in total As in the experimental and control groups after 6 h of reaction.

Ion chromatography was applied to analyze the post-reaction solutions. By comparing the retention times of standard and experimental samples, the presence of sulfate ions (SO_4_^2−^) in the solutions was successfully confirmed. Under the chromatography condition set, the retention time of SO_4_^2−^ was 10.847 min. Combining with the comparative data of the total sulfur content in the solution and the content of SO_4_^2−^ shown in Figure 3A, it was determined that sulfur in the reaction system mainly existed in the form of SO_4_^2−^.

In addition, the molybdenum blue spectrophotometric method was used for quantitative analysis of samples collected at 6 h of reaction. By comparing the absorbance changes before and after adding KMnO_4_ to oxidize As(III) in samples, the valence state distribution of arsenic in different reaction systems was clarified: in the ·OH experimental group, all arsenic elements in the solution after the reaction were converted into AsO_4_^3−^; in the only hν group, arsenic mainly existed in the form of arsenite ions (AsO_2_^−^); in the hydrogen peroxide group, arsenic predominantly existed as AsO_4_^3−^ (Figure 3B).

**Figure 4:**
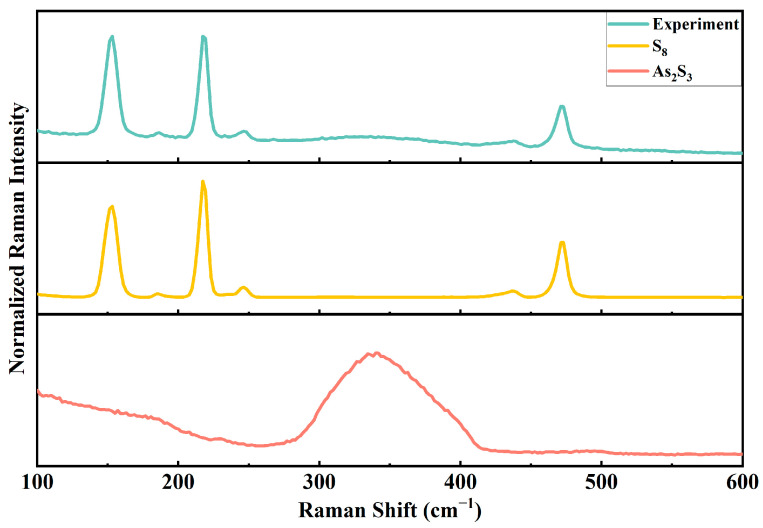
Raman spectra of the ·OH experimental group with S_8_ and As_2_S_3_.

Subsequently, the post-reaction precipitates were characterized. First, Raman spectroscopy was performed on the precipitates. As shown in Figure 4, by comparing the experimental results with the spectra of amorphous As_2_S_3_ and pure S_8_, distinct characteristic peaks of elemental sulfur were observed in the post-reaction precipitate at 153 cm^−1^, 219 cm^−1^, and 472 cm^−1^. Specifically, the peak at 153 cm^−1^ corresponds to the bending vibration of the S-S bond, the peak at 219 cm^−1^ corresponds to the symmetric stretching vibration, and the peak at 472 cm^−1^ corresponds to the torsional vibration. These characteristic peaks are highly consistent with the Raman vibrational features of elemental sulfur, confirming the formation of elemental sulfur in the post-reaction precipitate.

To determine the chemical composition of the precipitate formed after the reaction, X-ray Photoelectron Spectroscopy (XPS) analysis was performed on both the precipitates before and after the reaction. XPS spectra were obtained for pure orpiment and the solid samples resulting from the reaction between various reactive oxygen species and orpiment (Figures S4 and S5). The fine spectra of each element were charge-corrected using the binding energy of C 1s (284.8 eV), and background subtraction was performed using the Tougaard method. Subsequently, peak deconvolution processing was applied to the high-resolution spectra. The results were compared with data from the National Institute of Standards and Technology (NIST) X-ray Photoelectron Spectroscopy database to determine the chemical assignment of each characteristic peak.

**Figure 5:**
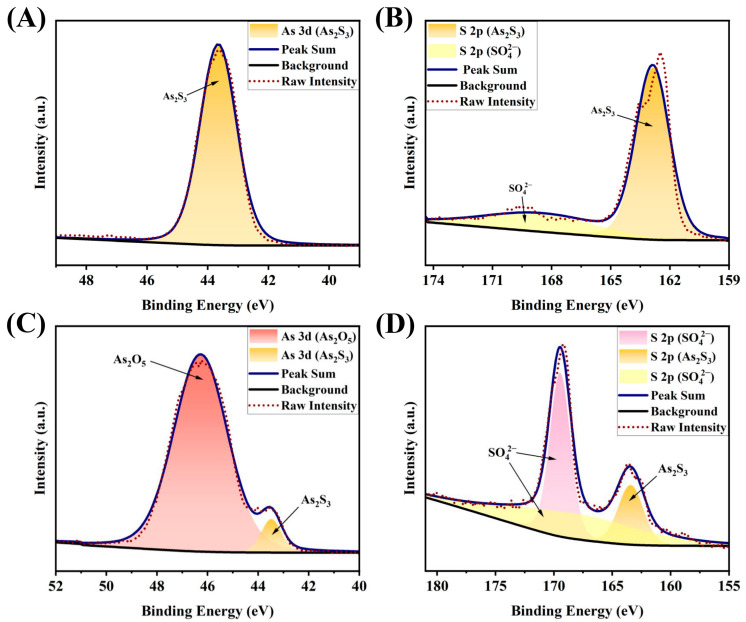
XPS spectra and peak results of raw materials As 3d (**A**), S 2p (**B**), and precipitates As 3d (**C**) and S 2p (**D**) after the ·OH reaction.

The high-resolution As 3d spectrum and peak deconvolution results for pure orpiment are shown in Figure 5A, where the peak at (43.4 ± 0.1) eV can be attributed to As_2_S_3_ [14]. Figure 5B shows the high-resolution S 2p spectrum and deconvolution results of pure orpiment. The peak at (163.4 ± 0.1) eV is the characteristic peak of As_2_S_3_, while the minor peak at (169.5 ± 0.1) eV indicates the possible presence of a small amount of sulfate compounds [15] in the orpiment raw material.

The high-resolution As 3d spectrum and peak deconvolution results of the sample after reaction with ·OH are shown in Figure 5C. The peak centered at (46.2 ± 0.1) eV corresponds to As_2_O_5_ [16], while the peak at (43.4 ± 0.1) eV is attributed to unreacted As_2_S_3_. Figure 5D shows the high-resolution S 2p spectrum and deconvolution results of the sample after the reaction. The characteristic peak at (169.5 ± 0.1) eV indicates the presence of sulfates, while the peak at (163.4 ± 0.1) eV originates from As_2_S_3_. Notably, no XPS characteristic peaks for elemental sulfur were detected, likely due to its extremely low concentration in the precipitate, causing the signal to be masked by other components.

Based on the comprehensive analysis of Raman spectroscopy and XPS characterization results, it is concluded that, besides the original As_2_S_3_, the post-reaction precipitate also contains As_2_O_5_, S_8_, and sulfate compounds.

### 2.3. Characterization of the Reaction Results of the Other ROS

Following the clarification of the reaction characteristics between orpiment and ·OH, the same characterization methods were employed to investigate the reaction processes and products of ^1^O_2_, ONOO^−^, and H_2_O_2_ with As_2_S_3_. The characterization data are presented in Figures S1–S3. The experimental results reveal a high degree of similarity in the reaction products of these three reactive oxygen species with orpiment: sulfur in the solution predominantly exists in the form of SO_4_^2−^; among the sulfur-containing compounds in the solid, in addition to the unreacted As_2_S_3_, sulfate substances and S_8_ were also detected. Regarding the speciation of arsenic, arsenic in the solution after the reaction mainly exists as AsO_4_^3−^, accompanied by a small amount of AsO_2_^−^; the arsenic-containing compounds in the solid are composed of As_2_S_3_ and arsenic trioxide (As_2_O_3_) [17].

### 2.4. Based on Gibbs Free Energy Change to Analyze Reaction Equations

The feasibility of various reactions was explored from a thermodynamic perspective by calculating the Gibbs free energy change (ΔG) of a series of equations for the reactions between orpiment and reactive oxygen species. First, as shown in Table 1, the reaction between orpiment and hydroxyl radicals was calculated. The results showed that the ΔG value of Equation (1) was the smallest, indicating that this reaction had the highest degree of spontaneity thermodynamically. In addition, the ΔG value of Reaction 3, which produced elemental sulfur, was also negative, suggesting that this reaction could proceed spontaneously under thermodynamic conditions. These theoretical calculation results were highly consistent with the experimental observations, where the main arsenic degradation product was AsO_4_^3−^, the main sulfur degradation product was SO_4_^2−^, accompanied by a small amount of elemental sulfur. It fully demonstrates that there are multiple pathways for the reactions between orpiment and ·OH, among which the reaction pathways leading to the formation of arsenate-based and sulfate-based compounds are dominant.

Subsequently, theoretical calculations were performed for the reactions of another three reactive oxygen species with orpiment using the same computational method. The results showed that theoretical simulations were in good agreement with the experimental data, and the Gibbs free energy changes of the reaction systems involving these three reactive oxygen species all exhibited negative values, indicating that the reactions are thermodynamically spontaneous (Tables S5–S7).

## 3. Discussion

**Figure 6:**
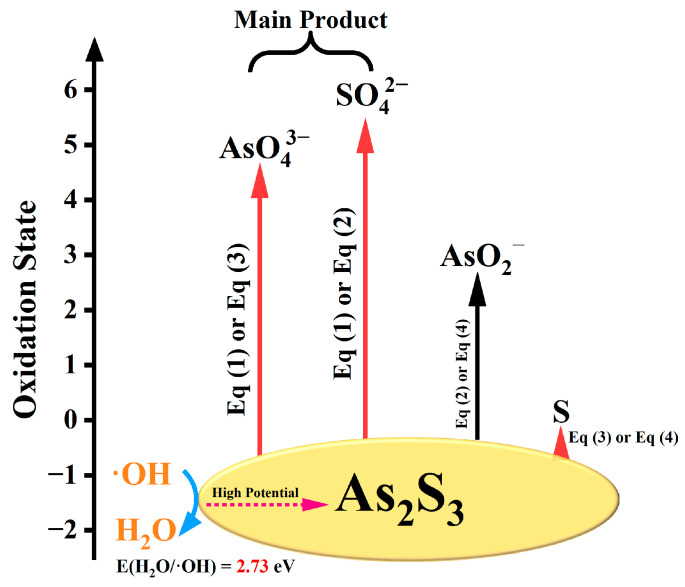
Schematic representation of the ·OH-mediated oxidation products of As_2_S_3_.

According to the experimental results from the reactions between four ROS and As_2_S_3_, S_8_ is preferentially formed due to the kinetic advantage of the reaction of S^2−^ oxidation to S_8_ (low reduction potential, E = −0.34 V). The products of the reaction between ·OH and As_2_S_3_ have the following two characteristics: the S-containing substances in the solution are almost SO_4_^2−^, and the dissolution amount of S element is much higher than that in the reactions involving other ROS; the dissolved As amount is the largest, and all As-containing substances are As (V). This is because the oxidation potential of ·OH is extremely high (~2.8 V), which can further oxidize the initially formed S_8_ to SO_4_^2−^, and oxidize As (III) released by the destruction of the lattice structure and disintegration of As_2_S_3_ to As (V), promoting the dissolution of As. Combining experimental observations with theoretical calculation results, we propose that the reaction of orpiment with hydroxyl radicals proceeds primarily via two pathways. Among them, Reaction (1) is the main reaction, while Reaction (3) is the secondary reaction. Inspired by Zhang’s work [18], we schematized the reaction as shown in Figure 6.

By contrast, the reactions of the other three ROS with As_2_S_3_ mainly focus on the “dissolution” of As_2_S_3_, i.e., relatively few valence changes occur. The vast majority of As involved in the reaction exists in the form of As_2_O_3_, and only part of the dissolved As (III) is oxidized; while the S^2−^ involved in the reaction is mainly converted to S^0^. This is because the oxidation potentials of ^1^O_2_, ONOO^−^, and H_2_O_2_ are relatively low, about 1.0~1.7 V, and the reaction with the lowest reduction potential (S^2−^→S^0^) is preferentially carried out. The resulting reaction product S^0^ exists in solid form, so the apparent dissolution efficiency of these three ROS for S is similar to that of some control groups. Given the relatively low oxidation potentials of these three reactive oxygen species, their oxidation capacity is insufficient to completely oxidize dissolved As (III) to As (V), which is manifested in the experimental observations as the reaction products predominantly consisting of As_2_O_3_ or AsO_2_^−^.

Based on the above results, we speculate that besides the photodegradation of As_2_S_3_, the long-term light irradiation generates a certain amount of reactive oxygen species in the air, which react with As_2_S_3_ to promote its degradation, producing substances such as As_2_O_3_ and As_2_O_5_. These arsenic oxides are white or grayish white, so the macroscopic manifestation is the fading of pigments. In addition, ions such as AsO_4_^3−^ and SO_4_^2−^ produced during the degradation process are prone to migrate in the pigment layer. If these ions come into contact with other pigments (such as Cu- and Pb-containing pigments), insoluble metal salts may be generated through reactions, which may macroscopically manifest as pigment discoloration. The stress generated during this process may also cause flaking, pulverization, and other phenomena of the pigments. However, the aging of orpiment occurs at the solid–liquid/solid–gas interface between the mural substrate and reactive oxygen species, whose reaction rate is significantly slower than in a solution system. To achieve long-term simulation of real-world conditions, further experiments will be required in the future.

## 4. Materials and Methods

### 4.1. Materials and Apparatus

The materials and apparatus were presented in Supplementary File (Tables S1 and S2).

### 4.2. Experimental Methods

#### 4.2.1. Reaction of As_2_S_3_ with ·OH

As_2_S_3_ (297 mg, 1.2 mmol) was added to a quartz glass conical flask containing 50 mL of ultrapure water under stirring, followed by full-spectrum irradiation using a mercury lamp (illumination power: 500 W). The reaction temperature was controlled at 25 °C, and 220 μL of H_2_O_2_ was intermittently added over 2.5 h. Samples were taken at 0 min, 30 min, 1 h, 2 h, 3 h, and 6 h intermittently. After 6 h, the light was turned off to terminate the reaction. With the same conditions, the control experiments were performed except for the following. For the only hν group, 220 μL of H_2_O_2_ was replaced with 220 μL of H_2_O; for the only H_2_O_2_ group, no lamp was used; for the inhibitor group, 50 mL of 10% isopropanol aqueous solution was used to replace the ultrapure water.

#### 4.2.2. Reaction of As_2_S_3_ with ^1^O_2_, ONOO^−^, or H_2_O_2_

For the experiments of As_2_S_3_ with ^1^O_2_, 297 mg of As_2_S_3_ was added to a quartz glass conical flask containing 7.65 mL of NaClO solution and 43.5 mL of ultrapure water. The mixture was kept in the dark, stirred, and the reaction temperature was controlled at 25 °C. Over 2.5 h, 220 μL of H_2_O_2_ was intermittently added. Samples were taken at 0 min, 30 min, 1 h, 2 h, 3 h, and 6 h. The reaction was terminated after 6 h. The details including control experiments are shown in Table 2.

For the experiments of As_2_S_3_ (297 mg) with ONOO^−^ or H_2_O_2_, the procedure is the same except for the different reagents listed in Table 2.

### 4.3. Sample Treatment Methods

The obtained samples were centrifuged, and the supernatant was aspirated using a 5 mL syringe, filtered through a syringe filter (MCE membrane, pore size 0.45 μm), and the resulting solution was stored in a refrigerator for further use.

The precipitates after centrifugation were pre-frozen at −20 °C and then lyophilized. The dried solids were ground, collected, and stored in a desiccator.

### 4.4. Sample Characterization

#### 4.4.1. Inductively Coupled Plasma Optical Emission Spectrometry (ICP-OES) Test

The supernatant sample (100 μL) was mixed with 500 μL of 1.0 M HNO_3_ solution and diluted to 10.0 mL with ultrapure water. ICP-OES was performed to determine the total contents of element As and S in the sample.

#### 4.4.2. Ion Chromatography (IC) Test

The ion chromatograph was equipped with a DS6 conductivity detector, AERS 4 mm anion suppressor, CERS 4 mm cation suppressor, Chromeleon 6.8 chromatography workstation, IonPac AS22 anion analysis column (250 mm × 4 mm), and IonPac AG22 anion guard column (50 mm × 4 mm). The test was conducted at a column temperature of 33 °C and a flow rate of 1.0 mL/min using a mixed buffer of 9.0 mM NaHCO_3_/0.8 mM Na_2_CO_3_ as the eluent.

SO_4_^2−^ and AsO_4_^3−^ standard solutions (each 200 μL of 1000 μg/mL) were separately diluted to 10.0 mL with ultrapure water for IC to determine the retention times and corresponding peak areas of the standards.

The supernatant sample (20 μL) was diluted to 10.0 mL with ultrapure water for IC testing, and the contents of SO_4_^2−^ and AsO_4_^3−^ were calculated based on the standard solution results.

#### 4.4.3. Molybdenum Blue Spectrophotometry Test

Determination of the relative contents of AsO_2_^−^ and AsO_4_^3−^ were performed as follows: The supernatant sample was diluted 100-fold, and 1000 μL of the diluted solution was mixed with 2 mL of 50% H_2_SO_4_ solution. The mixture was equally divided into two aliquots (A and B), and aliquot A was added with 0.001 M KMnO_4_ solution dropwise until slightly red. Then, both aliquots were added with 20 mL of ultrapure water, 2 mL of 3% ammonium molybdate solution, and 1 mL of 1% ascorbic acid solution, heated in a boiling water bath for 20 min, cooled, and diluted to 50 mL with ultrapure water. Absorbance was measured by UV-Vis absorption spectroscopy at 620 nm. The absorbance of group A corresponded to the total content of AsO_2_^−^ and AsO_4_^3−^, while that of group B corresponded to the content of AsO_4_^3−^.

#### 4.4.4. Raman Spectroscopy Test

Raman spectra of As_2_S_3_, S_8_, and the treated precipitates were scanned in the range of 100 cm^−1^–1000 cm^−1^ with a 532 nm He-Ne laser as an excitation source. The results were processed using LabSpec (version 6 6.6.1.11) software for baseline correction.

#### 4.4.5. X-Ray Photoelectron Spectroscopy (XPS) Test

XPS scans were performed on the treated precipitates to obtain full XPS spectra and high-resolution spectra of C 1s, S 2p, and As 3d by using Al Kα radiation. Data processing was carried out using CasaXPS (version 2.3.26) software: the high-resolution C 1s spectrum was peak-fitted to obtain the binding energy of C 1s for charge correction of the spectra. The corrected high-resolution S 2p and As 3d spectra were then peak-fitted, and the binding energies were compared with known values in the NIST X-ray Photoelectron Spectroscopy Database to determine the attribution of each peak.

#### 4.4.6. Quantum Chemical Computational Methods and Conditions

The computational methods and conditions used are as follows. All electronic calculations were performed using the Gaussian 16 software package. Quantum chemical calculations were carried out with the Gaussian 16 software package (Revision D.01) and GaussView 6.0, which was used to visualize the optimal structures and molecular frontier orbitals of the molecules involved in the reactions. The temperature was set to 298.15 K, the pressure to 1 atm, and the solvent model employed was IEFPCM with water as the solvent. Density functional theory (DFT) at the B3LYP/6-311G(d,p) basis set level was used to optimize the ground-state geometries of the molecules in the reactions. Frequency calculations and vibrational analyses were performed to ensure the absence of imaginary frequencies, thereby confirming the molecular configurations. The B3LYP/6-311G(d,p) basis set was also used for energy calculations.

## 5. Conclusions

This study is set against the backdrop of reactive oxygen species (ROS) widely existing in nature. Aiming at the phenomenon that the orpiment pigment layer of Dunhuang murals undergoes degradation of varying degrees over time, reactions between As_2_S_3_ and a series of ROS were designed. The reaction products were characterized, the reaction results of As_2_S_3_ with ROS were analyzed and interpreted, and a possible reaction mechanism was proposed. We speculate not only that the photodegradation of As_2_S_3_ does result from the direct decomposition of As_2_S_3_ under light exposure but also that long-term illumination generates a certain amount of ROS in the air. These ROS come into contact with the surface of the pigment layer through diffusion and react with As_2_S_3_ therein, leading to the degradation of As_2_S_3_. The proposal of this mechanism explains why the orpiment pigment layer not exposed to light still exhibits phenomena such as fading, which helps provide theoretical support for the protection of pigment layers and improve existing protection strategies.

## Figures and Tables

**Table 1:** Possible reaction pathways of As_2_S_3_ with ·OH and their Gibbs free energy changes.

Eqs	Reaction	ΔG/(kJ/mol)
(1)	As_2_S_3_ + 28·OH → 2H_3_AsO_4_ + 3H_2_SO_4_ + 8H_2_O	−4893
(2)	As_2_S_3_ + 24·OH → 2HAsO_2_ + 3H_2_SO_4_ + 8H_2_O	−4188
(3)	As_2_S_3_ + 10·OH → 2H_3_AsO_4_ + 3S + 2H_2_O	−785.7
(4)	As_2_S_3_ + 6·OH → 2HAsO_2_ + 3S + 2H_2_O	−80.48

**Table 2:** Reagent dosage configuration for sample and control groups in the reaction of As_2_S_3_ with different reactive oxygen species (^1^O_2_, ONOO^−^, H_2_O_2_).

Reagents		NaClO	NaNO_2_	H_2_O_2_	H_2_O
^1^O_2_	Sample	7.6 mL	-	220 μL	42.4 mL
	Control	7.6 mL	-	-	42.4 mL
ONOO^−^	Sample	-	158 mg	220 μL	50 mL
	Control	-	158 mg	-	50 mL
H_2_O_2_	Sample	-	-	220 μL	50 mL
	Control	-	-	-	50 mL

## Data Availability

The original contributions presented in this study are included in the article/Supplementary Materials.

## Supplementary Materials

The following supporting information can be downloaded at https://www.mdpi.com/article/10.3390/molecules30163318/s1.

## Abbreviations

The following abbreviations are used in this manuscript:

ROS	Reactive Oxygen Species
ICP-OES	Inductively Coupled Plasma Optical Emission Spectrometry
IC	Ion Chromatography
XPS	X-ray Photoelectron Spectroscopy
