# Supplementary material for: Mechanistic Study on Orpiment Pigment Discoloration Induced by Reactive Oxygen Species
Source: Molecules. 2025 Aug 8;30(16):3318. doi: 10.3390/molecules30163318 (PMC12388804; doi:10.3390/molecules30163318)
Supplement: Supplementary file 1 [file molecules-30-03318-s001.zip › molecules-3762215-supplementary.pdf]

*Supplementary Materials*

# Mechanistic Study on Orpiment Pigments Discoloration Induced by Reactive Oxygen Species

**Jiaxing Sun[1], Zhehan Zhang[1], Xiaofen Chen[1], Qin Huang[1], Zhilin Bian[1], Wenyuan Zhang[2], Bomin Su[2] and Haixia Zhang[1,*]**

[1] State Key Laboratory of Natural Product Chemistry, College of Chemistry and Chemical Engineering, Lanzhou University, Lanzhou 730000, China;

[2] Gansu Provincial Research Center for Conservation of Dunhuang Cultural Heritage, Dunhuang Academy, Dunhuang 736200, China

* Correspondence: zhanghx@lzu.edu.cn; Tel: +86-931-8912510

**Table S1.** Reagents Used in the Experiments.

| Reagent Name | Chemical Formula | Manufacturer |
|---|---|---|
| Orpiment | $As_2S_3$ | Anhui Zesheng Technology Co., Ltd. |
| Hydrogen Peroxide | $H_2O_2$ | Chengdu Kelong Chemicals Co., Ltd. |
| Isopropanol | $C_3H_8O$ | Tianjin Fuyu Fine Chemicals Co., Ltd. |
| Sodium Hypochlorite | $NaClO$ | Sain Chemical Technology (Shanghai) Co., Ltd. |
| Sodium Nitrite | $NaNO_2$ | Chengdu Kelong Chemicals Co., Ltd. |
| Pyrogallol | $C_6H_6O_3$ | Sain Chemical Technology (Shanghai) Co., Ltd. |
| Nitric Acid | $HNO_3$ | Baiyin Liangyou Chemical Reagent Co., Ltd. |
| Sodium Sulfate | $Na_2SO_4$ | Tianjin Damao Chemical Reagent Factory |
| Sodium Arsenate Dodecahydrate | $Na_3AsO_4 \cdot 12H_2O$ | Beijing Chemical Plant |
| Sulfuric Acid | $H_2SO_4$ | Chengdu Kelong Chemicals Co., Ltd. |
| Potassium Permanganate | $KMnO_4$ | Tianjin Guangfu Fine Chemical Research Institute |

| | | |
|---|---|---|
| Ammonium Molybdate Tetrahydrate | $(NH_4)_6Mo_7O_{24}·4H_2O$ | Tianjin Guangfu Technology Development Co., Ltd. |
| Ascorbic Acid | $C_6H_8O_6$ | Sinopharm Chemical Reagent Co., Ltd. |

**Table S2.** Instruments Required for Reactions and Characterization.

| Instrument Name | Model | Manufacturer |
|---|---|---|
| Analytical Balance | XS105DU | Mettler Toledo, Switzerland |
| Magnetic Stirrer | RCT basic | IKA, Germany |
| Mercury Lamp Light Source System | CEL-M500 | Beijing Zhongjiao Jinyuan Technology Co., Ltd. |
| High-Resolution Inductively Coupled Plasma Optical Emission Spectrometer | PQ9000 | Analytik Jena, Germany |
| Ion Chromatograph | Dionex ICS-1500 | Thermo Fisher Scientific (Dionex), USA |
| UV-Vis Spectrophotometer | T700 | Beijing Puxin General Instrument Co., Ltd. |
| Laser Confocal Raman Spectrometer | HR Evolution | HORIBA FRANCE SAS |
| X-ray Photoelectron Spectrometer | Axis Supra | Shimadzu, Japan |

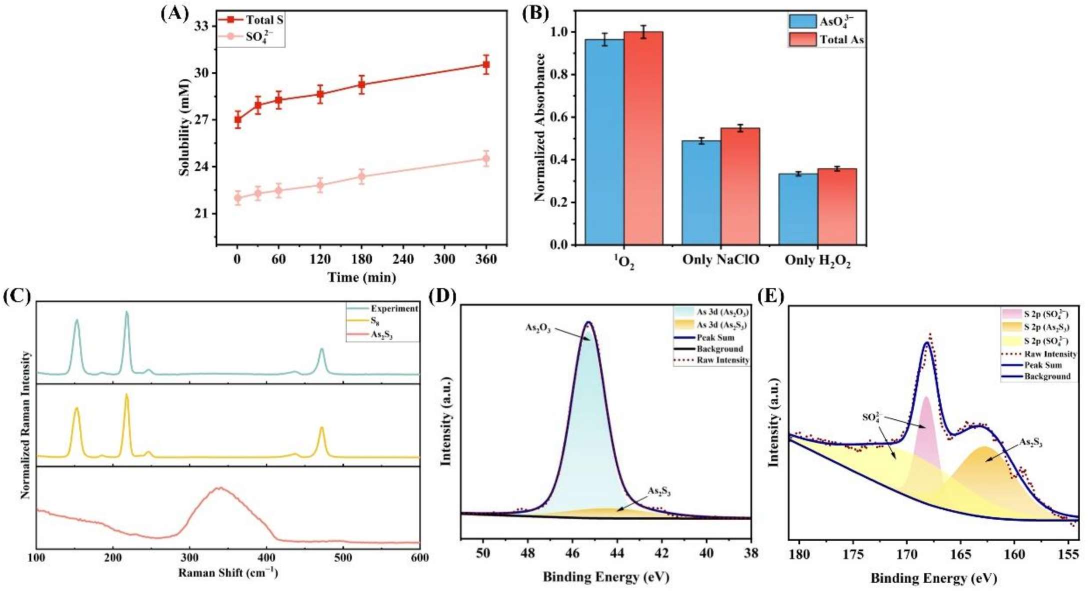


**Figure S1.** Characterization results of the reaction between $^1O_2$ and $As_2S_3$. (A) Concentration-time trends of total sulfur (Total S) and sulfate ions ($SO_4^{2-}$) in the solution; (B) Proportion of arsenate ions ($AsO_4^{3-}$) to total arsenic in the experimental and control groups after 6 hours of reaction; (C) Raman spectra of the solid products from the $^1O_2$ experimental group, compared with those of $S_8$ and $As_2S_3$; (D) X-ray photoelectron spectroscopy (XPS) and peak fitting results for As 3d in the solid product of the $^1O_2$-$As_2S_3$ reaction; (E) XPS and peak fitting results for S 2p in the same solid product.

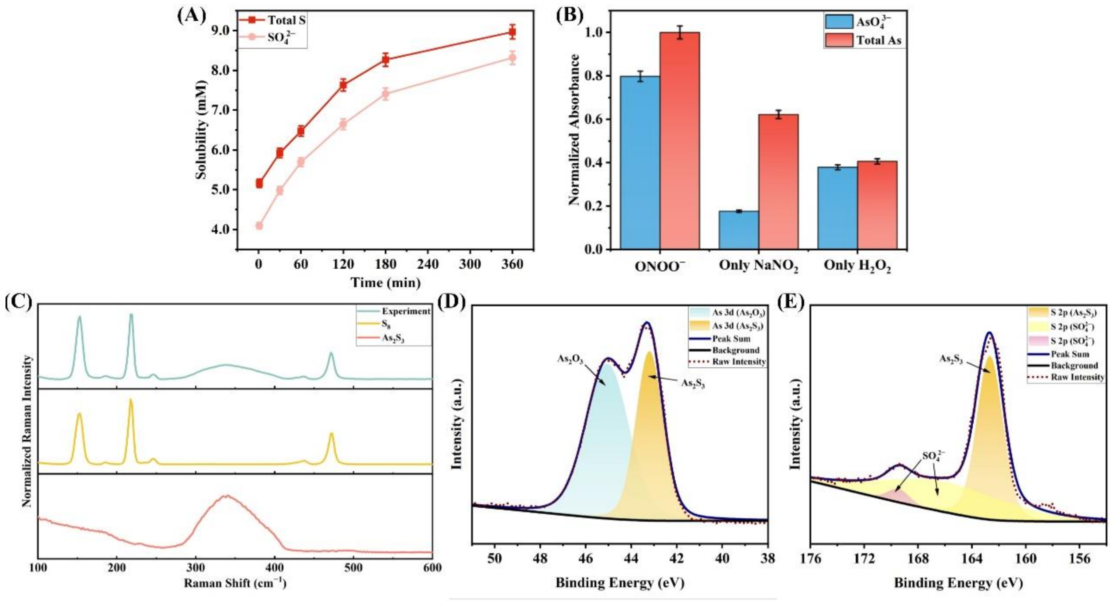


**Figure S2.** Characterization results of the reaction between $ONOO^-$ and $As_2S_3$. (A) Concentration-time trends of total sulfur (Total S) and sulfate ions ($SO_4^{2-}$) in the solution; (B) Proportion of arsenate ions ($AsO_4^{3-}$) to total arsenic in the experimental and control groups after 6 hours of reaction; (C) Raman spectra of the solid products from the $ONOO^-$ experimental group, compared with those of $S_8$ and $As_2S_3$; (D) X-ray photoelectron spectroscopy (XPS) and peak fitting results for As 3d in the solid product of the $ONOO^-$- $As_2S_3$ reaction; (E) XPS and peak fitting results for S 2p in the same solid product.

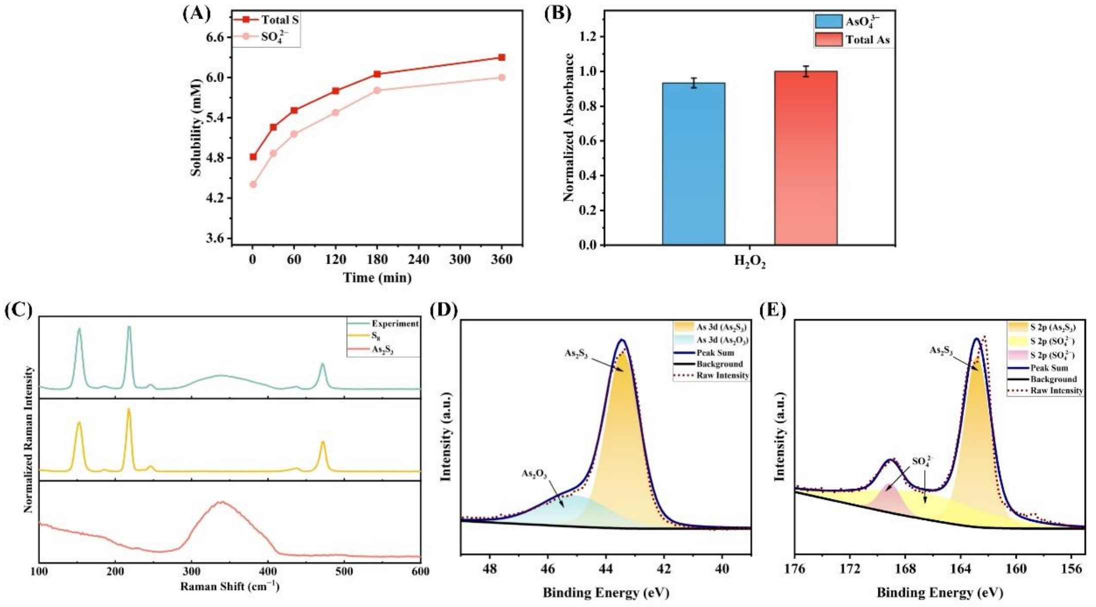


**Figure S3.** Characterization results of the reaction between $H_2O_2$ and $As_2S_3$. (A) Concentration-time trends of total sulfur (Total S) and sulfate ions ($SO_4^{2-}$) in the solution; (B) Proportion of arsenate ions ($AsO_4^{3-}$) to total arsenic in the experimental and control groups after 6 hours of reaction; (C) Raman spectra of the solid products from the $H_2O_2$ experimental group, compared with those of $S_8$ and $As_2S_3$; (D) X-ray photoelectron spectroscopy (XPS) and peak fitting results for As 3d in the solid product of the $H_2O_2$ $As_2S_3$ reaction; (E) XPS and peak fitting results for S 2p in the same solid product.

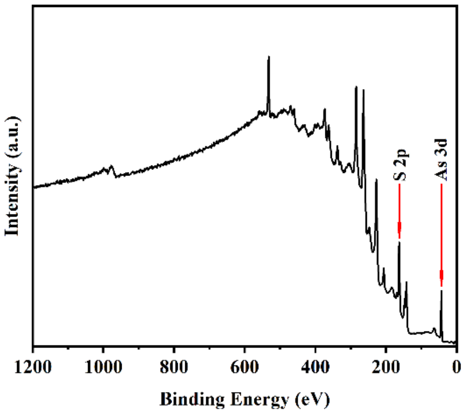


**Figure S4.** XPS survey spectrum of pure orpiment.

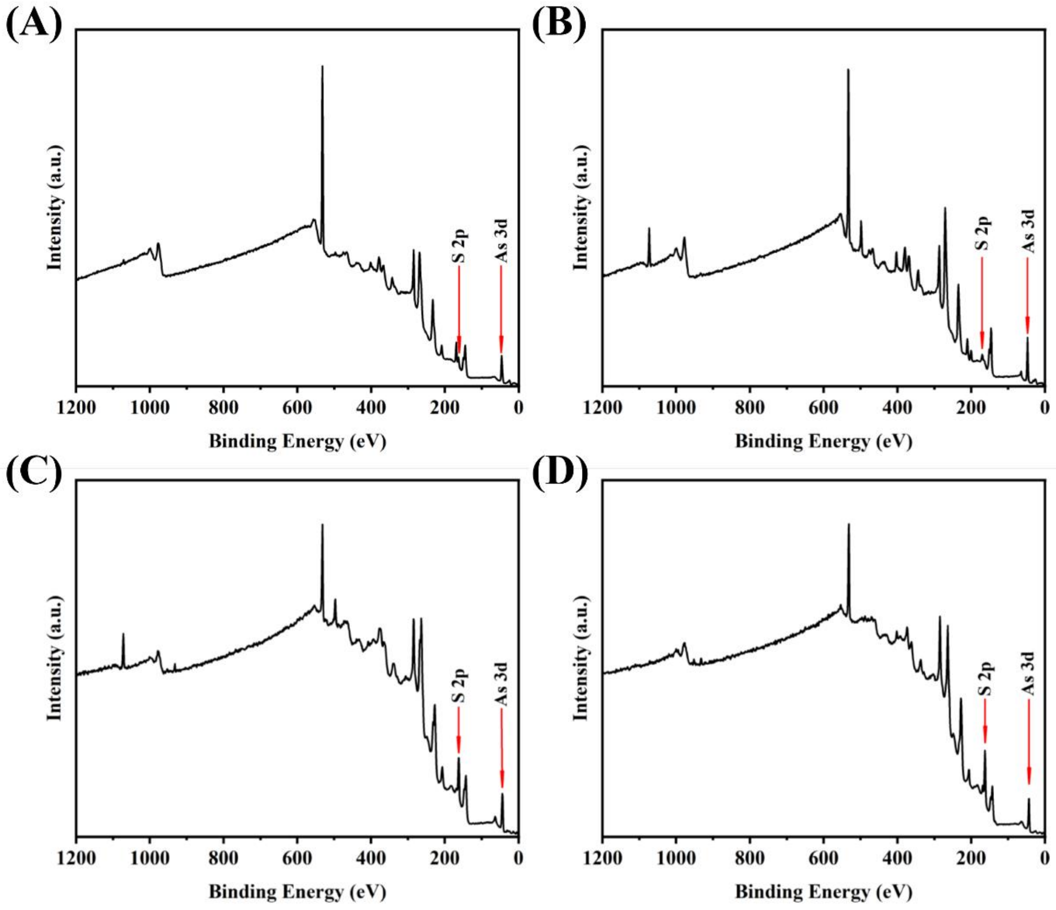


**Figure S5** XPS survey spectra of solid samples of orpiment after reaction with different reactive oxygen species. (A) ·OH group, (B) $^1O_2$ group, (C) $ONOO^-$ group, (D) $H_2O_2$ group.

**Table S3.** Details of Theoretical Calculation Parameters.

| **Parameter Category** | **Details** |
|---|---|
| Theoretical Approach | DFT |
| Type of Mission | Opt+Freq |
| Calculation Method | B3LYP |

| | |
|---|---|
| Basis Set | 6-311G (d, p) |
| Temperature | 298.15 K |
| Pressure | 1 atm |
| Solvation | Water |

**Table S4.** Calculated Energy Parameters for Compounds and Elements, all parameters are in Hartree units.

| Compound | EE | ZPE | ZPE+$\Delta U_{0\to298K}$ | ZPE+$\Delta H_{0\to298K}$ | ZPE+$\Delta G_{0\to298K}$ | U(0K) | U(298K) | H(298K) | G(298K) |
|---|---|---|---|---|---|---|---|---|---|
| $As_2S_3$ | −5666.49 | 0.00577 | 0.0127 | 0.0136 | −0.0268 | −5666.49 | −5666.48 | −5666.48 | −5666.52 |
| $H_2O$ | −76.4664 | 0.0212 | 0.0240 | 0.0250 | 0.00355 | −76.4452 | −76.4424 | −76.4414 | −76.4629 |
| $H_2O_2$ | −151.602 | 0.0264 | 0.0297 | 0.0306 | 0.00477 | −151.576 | −151.572 | −151.571 | −151.597 |
| $H_2S$ | −399.426 | 0.0150 | 0.0178 | 0.0188 | −0.00459 | −399.411 | −399.408 | −399.407 | −399.431 |
| $H_2SO_4$ | −700.353 | 0.0379 | 0.0432 | 0.0441 | 0.00955 | −700.316 | −700.310 | −700.309 | −700.344 |
| $H_3AsO_4$ | −2538.70 | 0.0438 | 0.0513 | 0.0522 | 0.0123 | −2538.66 | −2538.65 | −2538.65 | −2538.69 |
| $HAsO_2$ | −2386.99 | 0.0159 | 0.0197 | 0.0206 | −0.0109 | −2386.98 | −2386.97 | −2386.97 | −2387.00 |
| $NO_2^-$ | −205.225 | 0.00781 | 0.0107 | 0.0117 | −0.0159 | −205.217 | −205.214 | −205.213 | −205.241 |
| $^1O_2$ | −150.310 | 0.00370 | 0.00606 | 0.00701 | −0.0152 | −150.306 | −150.304 | −150.303 | −150.325 |
| ·OH | −75.7675 | 0.00842 | 0.0108 | 0.0117 | −0.00851 | −75.7591 | −75.7568 | −75.7558 | −75.7760 |
| $ONOO^-$ | −280.464 | 0.0106 | 0.0145 | 0.0154 | −0.0151 | −280.454 | −280.450 | −280.449 | −280.479 |
| S | −398.077 | 0 | 0.00142 | 0.00236 | −0.014896 | −398.077 | −398.076 | −398.075 | −398.092 |

The ΔG values for the following reactions are obtained by calculating the difference between the G values of the corresponding products and reactants, and then converting the units from Hartree to kJ/mol by equation S1.

$$1\,Hartree\ = 2625.5\ KJ/mol \qquad (S1)$$

**Table S5.** Possible Reaction Pathways of $As_2S_3$ with $^1O_2$ and Their Gibbs Free Energy Changes.

| Eqs | Reaction | ΔG/(kJ/mol) |
|---|---|---|
| 1 | $As_2S_3+7{}^1O_2+6H_2O\rightarrow 2H_3AsO_4+3H_2SO_4$ | −2196 |
| 2 | $As_2S_3+6{}^1O_2+4H_2O\rightarrow 2HAsO_2+3H_2SO_4$ | −1876 |
| 3 | $As_2S_3+4{}^1O_2+4H_2O\rightarrow 2H_3AsO_4+H_2SO_4+2S$ | −613.4 |

**Table S6.** Possible Reaction Pathways of $As_2S_3$ with $ONOO^-$ and Their Gibbs Free Energy Changes.

| Eqs | Reaction | ΔG/(kJ/mol) |
|---|---|---|
| 1 | $As_2S_3+14ONOO^-+6H_2O\rightarrow 2H_3AsO_4+3H_2SO_4+14NO_2^-$ | −527.9 |
| 2 | $As_2S_3+12ONOO^-+4H_2O\rightarrow 2HAsO_2+3H_2SO_4+12NO_2^-$ | −446.2 |
| 3 | $As_2S_3+11ONOO^-+5H_2O\rightarrow 2H_3AsO_4+2H_2SO_4+S+11NO_2^-$ | −94.12 |

**Table S7.** Possible Reaction Pathways of $As_2S_3$ with $H_2O_2$ and Their Gibbs Free Energy Changes.

| Eqs | Reaction | ΔG/(kJ/mol) |
|---|---|---|
| 1 | $As_2S_3+14H_2O_2\rightarrow 2H_3AsO_4+3H_2SO_4+8H_2O$ | −3232 |
| 2 | $As_2S_3+12H_2O_2\rightarrow 2HAsO_2+3H_2SO_4+8H_2O$ | −2764 |
| 3 | $As_2S_3+11H_2O_2\rightarrow 2H_3AsO_4+2H_2SO_4+S+6H_2O$ | −2218 |
